# MicroRNA-17-92 Regulates Beta-Cell Restoration After Streptozotocin Treatment

**DOI:** 10.3389/fendo.2020.00009

**Published:** 2020-01-23

**Authors:** Shan Wan, Jie Zhang, Xiang Chen, Jiangli Lang, Li Li, Fei Chen, Li Tian, Yang Meng, Xijie Yu

**Affiliations:** ^1^Laboratory of Endocrinology and Metabolism, Department of Endocrinology, National Clinical Research Center for Geriatrics, West China Hospital, Sichuan University, Chengdu, China; ^2^Histology and Imaging Platform, Core Facility of West China Hospital, Sichuan University, Chengdu, China; ^3^Department of Orthopedics, West China Hospital, Sichuan University, Chengdu, China

**Keywords:** miR-17-92 cluster, pancreatic beta-cells, streptozotocin, restoration, *Cdkn1a*, ATM kinase

## Abstract

**Objective:** To clarify the role and mechanism of miR-17-92 cluster in islet beta-cell repair after streptozotocin intervention.

**Methods:** Genetically engineered mice (*miR-17-92*βKO) and control RIP-Cre mice were intraperitoneally injected with multiple low dose streptozotocin. Body weight, random blood glucose (RBG), fasting blood glucose, and intraperitoneal glucose tolerance test (IPGTT) were monitored regularly. Mice were sacrificed for histological analysis 8 weeks later. Morphological changes of pancreas islets, quantity, quality, apoptosis, and proliferation of beta-cells were measured. Islets from four groups were isolated. MiRNA and mRNA were extracted and quantified.

**Results:** *MiR-17-92*βKO mice showed dramatically elevated fasting blood glucose and impaired glucose tolerance after streptozotocin treatment in contrast to control mice, the reason of which is reduced beta-cell number and total mass resulting from reduced proliferation, enhanced apoptosis of beta-cells. Genes related to cell proliferation and insulin transcription repression were significantly elevated in *miR-17-92*βKO mice treated with streptozotocin. Furthermore, genes involved in DNA biosynthesis and damage repair were dramatically increased in *miR-17-92*βKO mice with streptozotocin treatment.

**Conclusion:** Collectively, our results demonstrate that homozygous deletion of miR-17-92 cluster in mouse pancreatic beta-cells promotes the development of experimental diabetes, indicating that miR-17-92 cluster may be positively related to beta-cells restoration and adaptation after streptozotocin-induced damage.

## Introduction

Defective beta-cell function is one of the key reasons underlying the pathological process of both type 1 and 2 diabetes mellitus. Normal insulin-producing pancreatic beta-cells possess the powerful ability of adaptation and proliferation in response to chronic metabolic challenges such as obesity and gestation. For example, at the end of pregnancy, the beta-cell mass in normal rodents is increased by about 50% compared with non-pregnant female rodents (1, 2). Long-term high-fat diet feeding for 4 months also leads to a threefold increase in beta-cell mass and more insulin secretion in response to glucose stimulation (3). Individuals with the failed beta-cell function will gradually develop diabetes. Therefore, exploring the molecular and cellular mechanisms underlying the beta-cell adaptation and proliferation is critical for the intervention of diabetes.

Several studies have reported that microRNAs (miRNAs) were involved in the regulation of pancreatic beta-cell development, differentiation and insulin secretion (4–7). For example, Dicer1 governs the maturation of miRNAs, conditional deletion of which in mouse pancreas leads to abnormal development and differentiation of pancreatic cell lineages (8), indicating miRNAs are important for mouse pancreatic organogenesis. MiR-21, miR-29, miR-34a, miR-146, and miR-200a have been shown to be related to beta-cell apoptosis, whereas miR-7, miR-124a, miR-375, and miR-184 control insulin secretion (9–11). Furthermore, researchers have unveiled that the promoter region of miR-375 contains the binding domain of Pdx-1, Ngn3, and NeuroD1 which are pivotal transcriptional factors involved in beta-cell differentiation (12–15). Downregulation of miR-375 results in dedifferentiation of insulin secreting cells (16), whereas overexpression of miR-375 promotes the differentiation of pluripotent stem cells into beta-cell-like clusters (17). Intriguingly, overexpression of miR-375 together with downregulation of miR-9 show synergistic effects on the differentiation of human mesenchymal stem cells (hMSCs) into functional insulin-producing cells (18). Besides, miR-338-3p plays a key role in beta-cell adaptation to pregnancy and obesity, which is dramatically downregulated in rats islets at the 14th day of gestation (19). MiR-15a/b, miR-16, and miR-195 also show important roles in beta-cell development and specification (20). Moreover, overexpression of miR-124a2 leads to a decrease in several target genes such as Pdx-1, Kir6.2, and Sur-1, which are involved in glucose metabolism and insulin secretion (21).

Recent studies suggest that miR-17-92 cluster is also involved in islet beta-cell differentiation and development. Patients with gestational diabetes mellitus showed a high level of miR-17-92 cluster in plasma (22). In addition, the miR-17-92 cluster especially miR-17 was dramatically up-regulated in MIN6 cells and mouse islets after high glucose stimulation (23). When the nutrient substance was transited from fatty milk to full of carbohydrate diet at weaning, miR-17-92 cluster was down-regulated in rodent islets (24). MiR-18a inhibits pancreatic progenitor proliferation by suppressing the activation of proliferation-related signaling pathways phosphatidylinositol 3-kinase-protein kinase B (PI3K/AKT) and extracellular signal-regulated kinase (ERK) (25). MiR-19a promotes beta-cell proliferation and insulin secretion, while suppresses pancreatic beta-cell apoptosis through suppressor of cytokine signaling 3 (SOCS3), a direct target gene of miR-19a (26). Furthermore, the expression of miR-19b-1 in pancreatic progenitor cells is high, miR-19b-1 directly binds to the 3′UTR of NeuroD1 mRNA to downregulate its transcription, thus reduces the expression of insulin 1 gene, and consequently alters beta-cell differentiation and function (27). MiR-20a is upregulated in the peripheral blood mononuclear cells from type 1 diabetes patients (28). Overexpression of miR-92a-1 reduced insulin expression, in contrast, down-regulation of miR-92a-1 promoted insulin expression and ultimately enhanced glucose-induced insulin secretion (29). In addition, our previous studies have demonstrated the highest expression levels of miR-17 and miR-92a-1 in mouse pancreatic beta-cells, followed by miR-19b-1, miR-19a, miR-18a, and miR-20a (30). Furthermore, our studies suggested conditional knockout of miR-17-92 cluster in mouse pancreatic beta-cells impaired glucose tolerance and the first phase insulin secretion during intraperitoneal glucose tolerance test (IPGTT), which was further deteriorated by chronic high-fat diet feeding (30), suggesting that miR-17-92 cluster may be involved in the adaptation and proliferation of pancreatic beta-cells in response to chronic metabolic challenges.

Previous researches have shown that miR-17-92 was involved in regulation of development of multiple organs including heart, lung, lymphatic system, and reproductive system through targeting transcription factors that regulate cell proliferation and cell cycle and inhibiting the expression of apoptosis-related proteins (31, 32). However, little research has been done on its modulation of pancreatic beta-cell function. Because the function of beta-cell determines the development of diabetes, it is important to determine whether homozygous deletion of miR-17-92 cluster aggravates beta-cell dysfunction after streptozotocin treatment. In the current study, we used mice with conditional deletion of miR-17-92 cluster in islet beta-cells to investigate the role of miR-17-92 cluster in beta-cell adaptation as well as regeneration.

## Materials and Methods

### Experimental Animals

All animal procedures were approved by the Institute's Animal Care and Use Committee of the West China Hospital and followed the Guide for the care and use of laboratory animals. The rat insulin promoter Cre mice (RIP-Cre) (33) and the miR-17-92^flox/flox^ mice (34) both derived from C57BL/6J backgrounds, were acquired from the Jackson Laboratory (Bar Harbor, Maine, USA). RIP-Cre mice were mated with miR-17-92^flox/flox^ mice to generate experimental male mice with miR-17-92 cluster conditional deletion in pancreatic beta-cells (*miR-17-92*βKO mice). The RIP-Cre male mice were used as the control. All mice were maintained in a standard light-dark cycle and provided with free access to water and a standard diet.

### Streptozotocin Treatment

Male *miR-17-92*βKO and RIP-Cre mice at 12–16-week-old were injected with STZ (Sigma, Lot# WXBC2544V, P-Code: 101809717, USA) intraperitoneally for 5 consecutive days. The control mice of both genotypes were injected with citrate buffer. The STZ was dissolved in citrate buffer (PH = 4.5) at a dose of 50 mg/kg body weight. Random blood glucose (RBG) was measured at the third, seventh, and ninth day after the last STZ intervention. The mouse with RBG exceeding 16.7 mmol/l was recognized as a successful diabetic model, or the mouse was excluded from the diabetic groups. Body weight was measured once per week. The blood glucose levels after 16 h of fasting were recognized as fast blood glucose (FBG) and were tested once per week. RBG was monitored twice per week at 9 a.m. or 2 p.m.

### Intraperitoneal Glucose Tolerance Test

IPGTT was performed every week after STZ intervention, and total eight times were performed for each group. Glucose (2.0 g/kg body weight) was intraperitoneally injected after 16 h fasting. Blood was collected from the tail vein and blood glucose was measured with the Accu-Check glucometer (Roche, Indianapolis, IN) at 0, 15, 30, 60, and 120 min post-injection.

### Islets Isolation and Beta-Cell Sorting

The mice were sacrificed for histologic analysis 8 weeks after STZ intervention. The pancreatic islets isolation was performed as previously reported (35, 36). In short, collagenase P (Roche, Lot# 11036922, Germany) was dissolved in pre-cooling Hank's balanced salt solution without magnesium and calcium (Solarbio, Cat. No. H1045-500, Beijing, China) at a concentration of 1 mg/ml, and then the solution was retrogradely poured into the common bile duct. Subsequently, the pancreas was excised and digested in the 37°C water bath for 15 min. Finally, the islets were purified by density gradient along with hand sorting using an islet-specific coloring agent-dithizone (DTZ) (Shanghai Ryon Biological Technology CO., Ltd, Lot. RS31081B032, Shanghai, China).

### RNA Extraction and Quantitative RT-PCR Analysis

Total RNA was extracted from mice islets using Trizol reagent (Invitrogen, Bleiswijk, Netherlands), and both miRNA and mRNA were reversely transcribed into cDNA with PrimeScriptTM RT reagent kit (TaKaRa Biotechnology Co., Ltd., Dalian, China). Following reverse transcription, the cDNA was amplified and quantified with SYBR Green premix kit (TaKaRa Biotechnology Co., Ltd., Dalian, China) on Light Cycler 96 system (Roche, Basel, Switzerland). GAPDH and U6 were used as endogenous control for mRNA and miRNA, respectively. Each sample was conducted in triplicate and was analyzed with the 2^−ΔΔCT^ method.

### Pancreatic Histology and Immunofluorescent Staining

Mice pancreatic tissues were fixed in 10% paraformaldehyde solution and then embedded in paraffin. Pancreatic tissue blocks were further sectioned into 5 mm slices as previously reported (37). H&E (hematoxylin-eosin) staining was performed for histomorphological analysis.

Immunofluorescent staining was performed to evaluate the expression and location of insulin and glucagon using anti-insulin (cat# 8138, Cell Signaling Technology) and anti-glucagon (cat# sc-13091, Santa Cruz) antibodies. For BrdU labeling, mice were subcutaneously injected with BrdU (cat# B8010, Solarbo) dissolved in saline at a dose of 50 mg/kg body weight once a day for 4 days before sacrifice. The slices were successively incubated with anti-insulin and anti-BrdU (cat# sc-32323, Santa Cruz) antibodies then counterstained with DAPI (cat# 4083, Cell Signaling Technology) (38). The TUNEL assay kit (cat# G3250, Promega) was used to detect the apoptosis of pancreatic beta-cells. The LCA software was used to calculate pancreatic alpha-cell and beta-cell fraction automatically. The beta-cell mass per pancreas was calculated by multiplying the beta-cell fraction by the initial wet pancreatic weight. All data were obtained from at least three mice in each genotype as previously reported (15, 39).

### Statistical Analysis

SPSS 22.0 was used for data analysis. All data were shown as the mean ± standard error of the mean (SEM). The mRNA data were normalized to control conditions and presented as the relative expression. Multiple groups' comparison was achieved by one-way ANOVA. The Fisher's PLSD *post hoc* test was further conducted if there was a significant difference. Two-way ANOVA was used to detect the interactions between genotype and STZ treatment. The significant difference was set at *P* < 0.05 (two tails).

## Results

### The miR-17-92 Cluster Is Induced by Streptozotocin

**Figure 1:**
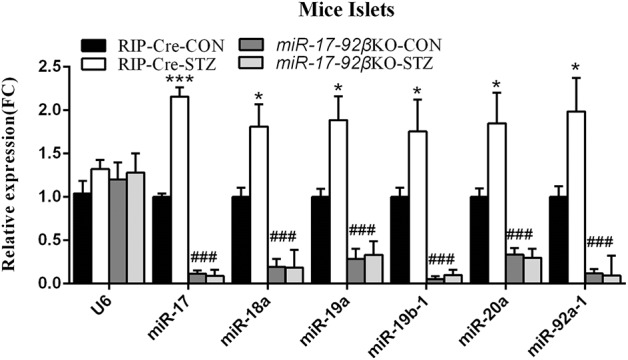
MiR-17-92 changes after streptozotocin treatment. Quantitative RT-PCR revealed that expression levels of miR-17-92 cluster significantly up-regulated in the isolated pancreatic islets from control mice but not *miR-17-92*βKO mice after STZ intervention. Statistical significance was shown as *P < 0.05, ***P < 0.001 compared to the same genotype, ###*P* < 0.001 compared to the same treatment. *N* = 16–20 mice/group.

As reported before, all members of the miR-17-92 cluster especially miR-17 and miR-92a-1 were highly expressed in mouse pancreatic islets and beta-cell line (30). We further studied the effect of STZ intervention on the expression of the miR-17-92 cluster in mouse islets. When compared to control mice treated with citrate buffer, control mice treated with STZ exhibited an increase in miR-17 expression by 116%, in miR-18a by 81%, in miR-19a by 89%, in miR-19b-1 by 76%, in miR-20a by 85%, in miR-92a-1 by 99% in pancreatic islets, respectively (Figure 1), suggesting that miR-17-92 cluster might play a key role in STZ-induced beta-cell damage and repair. However, *miR-17-92*βKO mice given either intervention (citrate buffer or STZ) showed similar few expression levels of miR-17-92 cluster in islets (Figure 1), indicating successful miR-17-92 cluster deletion in mouse pancreatic beta-cells.

### MiR-17-92 Homozygous Deletion in Mouse Pancreatic Beta-Cells Promotes Streptozotocin-Induced Metabolic Abnormities

**Figure 2:**
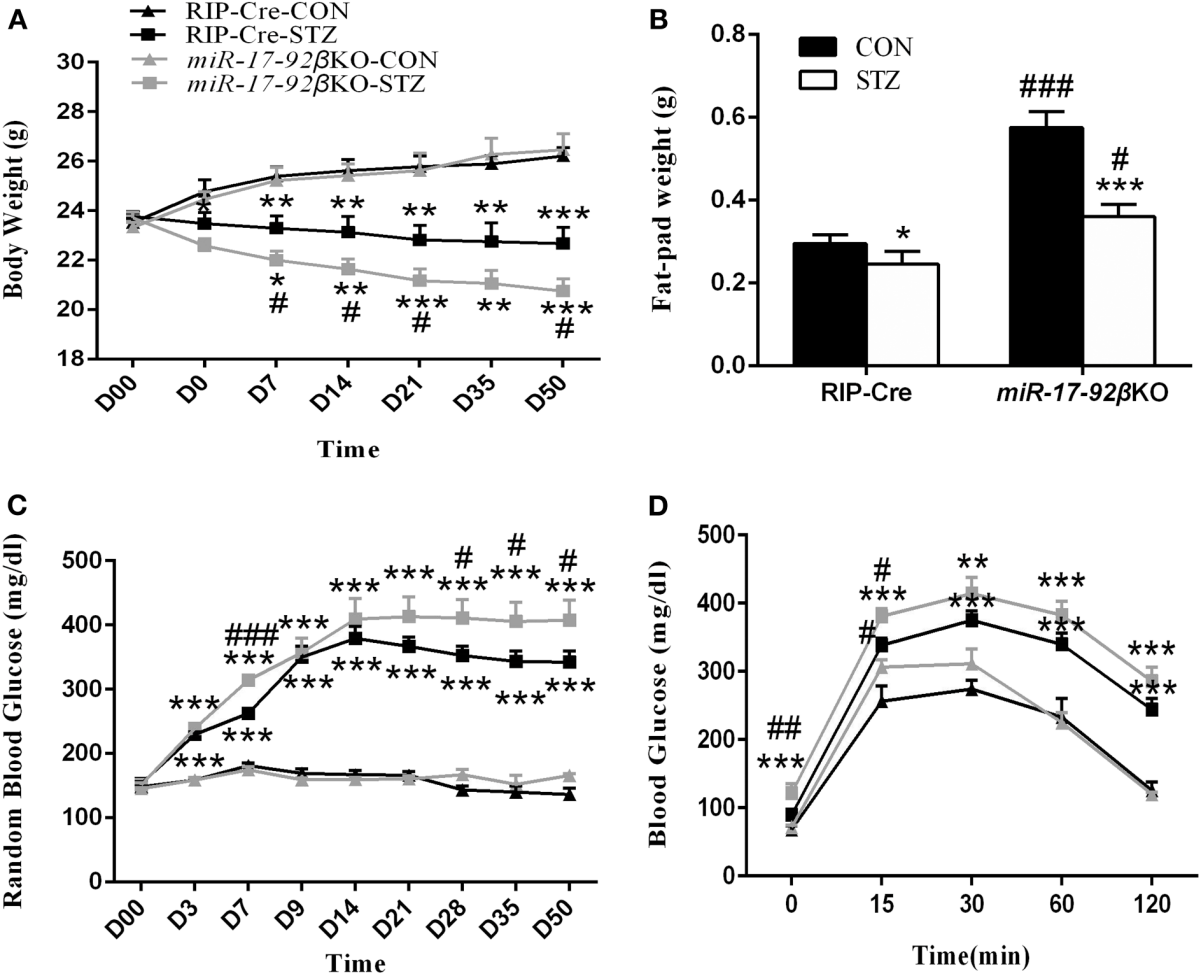
MiR-17-92 homozygous deletion in mouse pancreatic beta-cells promotes streptozotocin-induced metabolic abnormities. **(A–D)** Metabolic profiles of RIP-Cre and *miR-17-92*βKO mice treated with citrate buffer or STZ. Differences between control and *miR-17-92*βKO mice, including body weight **(A)**, fat-pad weight **(B)**, RBG **(C)**, and blood glucose levels of IPGTT **(D)**. Significant differences were shown as *P < 0.05, **P < 0.01, ***P < 0.001 compared to the same genotype, #*P* < 0.05, ##*P* < 0.01, ###*P* < 0.001 compared to the same treatment. *N* = 6–8 mice/group. “D00” and “D0” in **(A)** indicated the initial body weight of all mice and the body weight of mice after 5 consecutive day intraperitoneal STZ injection, respectively. The “D3” indicated the body weight of the third day of mice after 5 consecutive day intraperitoneal STZ intervention, so did the other related labels.

To investigate the pathophysiologic roles of the miR-17-92 cluster during type 1 diabetes development, we treated RIP-Cre and *miR-17-92*βKO mice with MLD-STZ. The MLD-STZ is proved to induce hyperglycemia through direct beta-cell DNA damage or indirect inflammatory response that leads to beta-cell dysfunction even death (40). In the present study, we treated 12 to 16-week-old male RIP-Cre and *miR-17-92*βKO mice with MLD-STZ and then performed metabolic and histological analysis on various time points after STZ intervention. Data showed that intraperitoneal injection of STZ for 5 consecutive days resulted in gradually body weight reduction in both RIP-Cre and *miR-17-92*βKO mice with the progress of the study. Especially, *miR-17-92*βKO mice injected with STZ exhibited more serious body weight loss than counterpart RIP-Cre-STZ mice, and at the end of the observation, the body weight of mice in RIP-Cre-STZ and *miR-17-92*βKO-STZ group reduced by 3.6 ± 0.3 g (13%) and 5.7 ± 0.5 g (22%) compared to RIP-Cre-CON and *miR-17-92*βKO-CON group, respectively (Figure 2A). The epididymal fat of *miR-17-92*βKO-CON group mice was higher than that of RIP-Cre-CON group. However, the epididymal fat pad of both genotypes was significantly reduced by 0.05 ± 0.02 g (17%) and 0.216 ± 0.03 g (38%), respectively, after STZ intervention (Figure 2B).

Additionally, before STZ intervention, the levels of RBG in four groups were similar. Whereas, the levels of RBG in both genotypes began to increase significantly since the third day after STZ injection. At the end of the experimental observation, the levels of RBG in RIP-Cre-STZ and *miR-17-92*βKO-STZ group increased by 206 ± 9.6 mg/dl (121%) and 242 ± 2.4 mg/dl (146%), respectively (Figure 2C). Meanwhile, *miR-17-92*βKO-STZ mice exhibited fasting hyperglycemia in contrast to RIP-Cre-STZ mice (121 ± 14.4 mg/dl vs. 90 ± 2.7 mg/dl; *P* < 0.05). When challenged with IPGTT, the changes in blood glucose in the *miR-17-92*βKO-CON group was similar as those in the RIP-Cre-CON group, which increased at 15, 30, and 60 min, but recovered to the fasting level at 120 min (Figure 2D, result at the sixth week; similar to other weeks, data not shown), indicating the glucose tolerance in the *miR-17-92*βKO-CON group was still in the compensatory state. Nevertheless, after STZ treatment, both genotypes of mice showed remarkable glucose intolerance. At the first IPGTT test, RIP-Cre-STZ group and *miR-17-92*βKO-STZ group began to exhibit elevated blood glucose levels at all time points (data not shown), the most obvious of which were in *miR-17-92*βKO-STZ group at the sixth IPGTT test with an 35, 13, 10, 13, and 17% elevation in 0, 15, 30, 60, and 120 min in contrast to the RIP-Cre-STZ group (Figure 2D).

Along with the research, the blood glucose levels of two genotypes of mice with STZ treatment restored slightly, but at the end of the experiment, the blood glucose levels were still higher than that of the CON mice, and the highest was in the *miR-17-92*βKO-STZ group. Taken together, these results suggest that mice with conditional deletion of the miR-17-92 cluster in islet beta-cells can physiologically maintain normal glucose metabolism homeostasis through the compensatory mechanism. However, when given exogenous stimulation (such as IPGTT or STZ-induced pancreatic beta-cell damage), the mice will lose its compensation, displaying beta-cell dysfunction, which indicates that the miR-17-92 cluster is required for the beta-cell function to STZ-induced damage.

### Reduced Proliferation and Elevated Apoptosis of Beta-Cells in *miR-17-92β*KO Mice Treated With Streptozotocin

**Figure 3:**
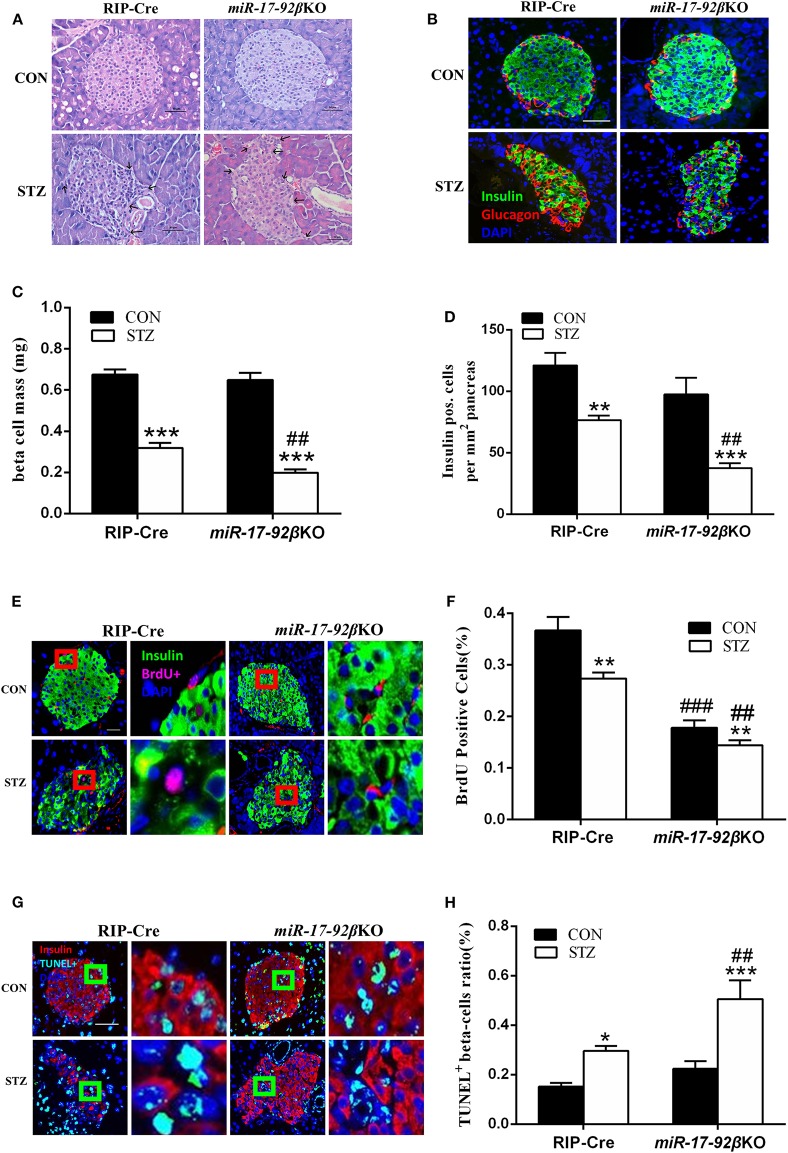
Reduced proliferation and elevated apoptosis of beta-cells in *miR-17-92*βKO mice treated with streptozotocin. **(A)** Microscopic photographs of islets from control and *miR-17-92*βKO mice after citrate buffer or STZ intervention, H&E staining, and original magnification 200×. Black arrows represent the abnormal islet morphology in mice treated with STZ. **(B)** Immunofluorescence staining for insulin (green), glucagon (red), and DNA (DAPI-blue) of islets from two genotypes, and original magnification 200×. **(C,D)** Quantitation of pancreatic islets beta-cell mass **(C)** and insulin positive cells per mm^2^ pancreas **(D)** of control and *miR-17-92*βKO mice after different treatments. **(E–H)** Analysis of BrdU positive cells (% of insulin-positive beta-cells) **(E,F)** and TUNEL positive beta-cells ratio (%) **(G,H)** by immunofluorescence staining of insulin-positive beta-cells in pancreatic islets from both genotypes. The pictures on the right are the corresponding enlarged pictures of the red/green box in the left pictures. Significant differences were shown as *P < 0.05, **P < 0.01, ***P < 0.001 compared to the same genotype, ##*P* < 0.01, ###*P* < 0.001 compared to the same treatment. *N* = 16–20 mice/group. Scale Bar = 50 μm.

In order to further confirm whether the miR-17-92 cluster modulates beta-cell regeneration after MLD-STZ intervention, we performed histomorphological analyses of pancreatic islets from the four groups. The pancreatic islets from four groups were first analyzed by H&E staining. Compared with RIP-Cre-CON mice, the islets of *miR-17-92*βKO-CON mice showed normal morphology, complete structure, and distinct division of cytoplasm and nucleus (Figure 3A), while the islets of both genotypes were significantly impaired after STZ intervention, manifested by abnormal islet morphology, scattered structure, and increased peri-islet neovascularization, which was more serious in *miR-17-92*βKO-STZ mice (Figure 3A).

Then, the pancreatic islets were analyzed by immunofluorescent staining. Compared to mice treated with citrate buffer, mice treated with STZ showed dramatically reduced insulin-positive and total mass of pancreatic beta-cells, and the distribution of alpha-cells changing from peripheral to scattered, among which the total mass of beta-cells declined by 38%, and the insulin-positive beta-cells decreased by 51% in islets from *miR-17-92*βKO-STZ mice in contrast to the RIP-Cre-STZ mice, suggesting damaged beta-cell regeneration in mice with miR-17-92 homozygous deletion in beta-cells after MLD-STZ treatment (Figures 3B–D). To clarify whether the damaged regeneration resulted from impaired beta-cell proliferation, we further conducted BrdU-insulin immunofluorescent staining to access beta-cell mitotic rate. Data showed that the proliferation of beta-cells diminished in *miR-17-92*βKO-CON mice compared to RIP-Cre-CON mice (Figures 3E,F). In addition, the proliferation of islet beta-cells further decreased by 47% in *miR-17-92*βKO-STZ group compared with RIP-Cre-STZ group (Figure 3F). Interestingly, we carried out TUNEL-insulin immunofluorescent staining to evaluate the apoptosis of the beta-cells and found that the apoptosis increased by 71% in the *miR-17-92*βKO-STZ group on contrast to the RIP-Cre-STZ mice (Figures 3G,H). Collectively, the decreased proliferation and elevated apoptosis reflect declining regeneration and conversion capacity of islet beta-cells in response to STZ-induced damage.

### The miR-17-92 Cluster Regulates Beta-Cell Number and Function

**Figure 4:**
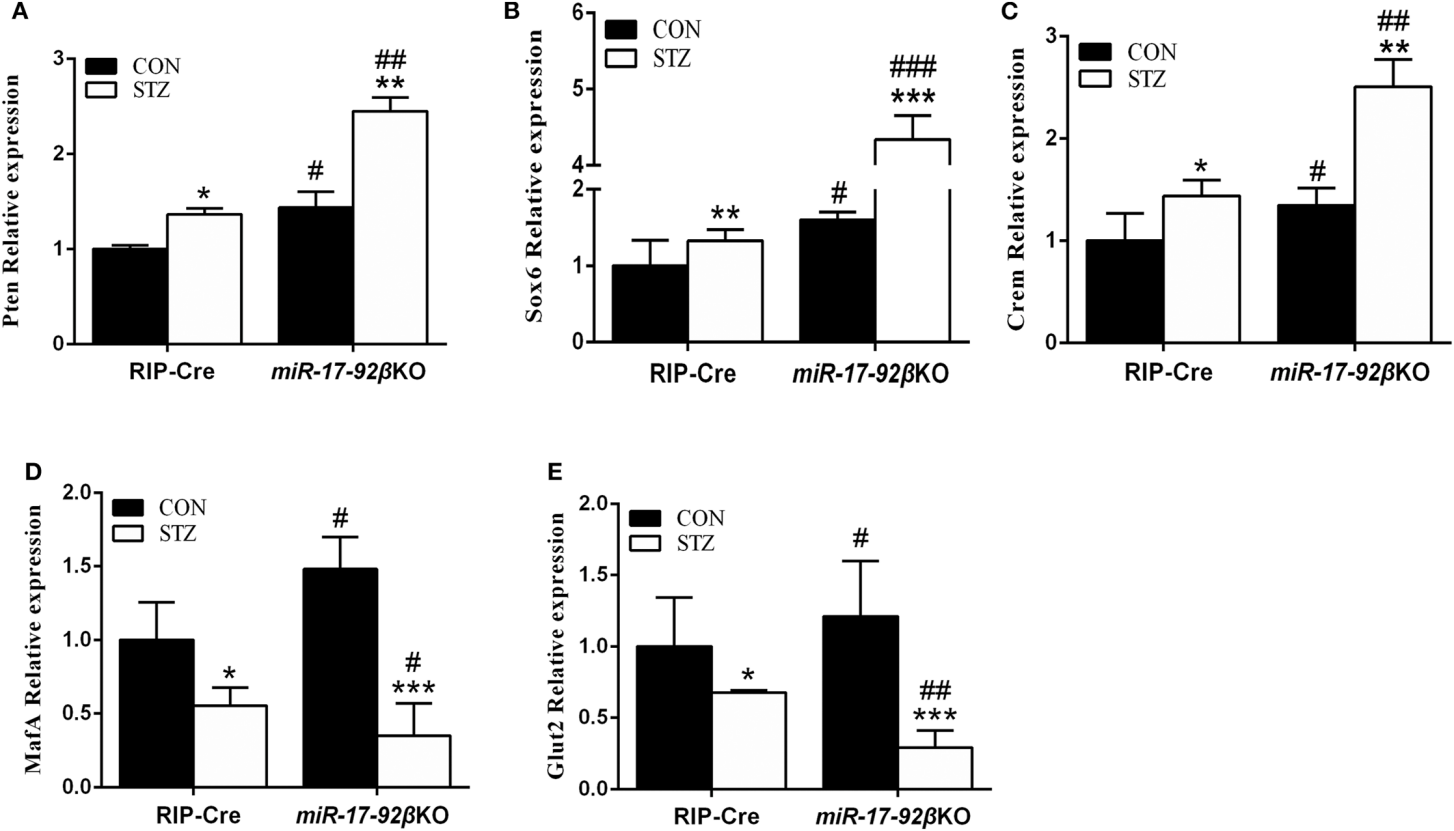
Impaired signaling pathways of pancreatic beta-cells' proliferation and insulin gene transcription. **(A)** Quantitative RT-PCR demonstrated that expression level of *Pten* significantly up-regulated in isolated islets from *miR-17-92*βKO mice treated with STZ. **(B,C)** Expression levels of Sox6 and Crem dramatically elevated in islets from *miR-17-92*βKO-STZ mice. **(D,E)** Expression levels of MafA and Glut2 remarkably decreased in islets from *miR-17-92*βKO mice given STZ treatment. Significant differences were shown as *P < 0.05, **P < 0.01, ***P < 0.001 compared to the same genotype, #*P* < 0.05, ##*P* < 0.01, ###*P* < 0.001 compared to the same treatment. *N* = 16–20 mice/group.

In order to further clarify the molecular mechanisms, we focused on the validated target genes of the miR-17-92 cluster involved in beta-cell proliferation and apoptosis. Compared with RIP-Cre-CON mice, mRNA expression of *Pten* (phosphatase and tensin homolog deleted on chromosome ten) in islets from *miR-17-92*βKO-CON mice was up-regulated by 44% (Figure 4A). After STZ intervention, mRNA expression of *Pten* was up-regulated by 36% in RIP-Cre-STZ group and 70% in the *miR-17-92*βKO-STZ group, respectively (Figure 4A), which may be the reason for the declining number and total mass of islet beta-cells in *miR-17-92*βKO-STZ mice.

Furthermore, the expression of genes related to insulin biosynthesis and secretion was further studied in islets from four groups of mice. Compared to RIP-Cre-CON mice, mRNA expressions of Sox6 (Sex-determination region Y-box 6) and Crem (cAMP response element modulator), genes related to insulin synthesis inhibition, up-regulated by 60 and 35% in islets of *miR-17-92*βKO-CON mice. The mRNA expressions of MafA (Mus musculus v-maf musculoapontic fibrocoma oncogene protein A) and Glut2 (Glucose transporter 2), genes related to insulin synthesis activation, in islets of *miR-17-92*βKO-CON mice were also up-regulated by 48 and 21% (Figures 4B–E). The mRNA expressions of Sox6 and Crem up-regulated by 32 and 44%, while mRNA expressions of MafA and Glut2 down-regulated by 45 and 32%, respectively in RIP-Cre-STZ mice (Figures 4B–E). At the same time, mRNA expression levels of Sox6 and Crem dramatically elevated by 171 and 86%, while mRNA expressions of MafA and Glut2 down-regulated by 76 and 75% separately in islets from *miR-17-92*βKO mice treated with STZ, resulting in inhibition of insulin transcription pathway in *miR-17-92*βKO-STZ mice (Figures 4B–E). Collectively, these results suggest that the miR-17-92 cluster is crucial for the quantity maintenance and insulin-producing function of the beta-cells during STZ treatment.

### Homozygous Deletion of the miR-17-92 Cluster in Beta-Cells Suppresses DNA Biosynthesis but Promotes DNA Damage Repair

**Figure 5:**
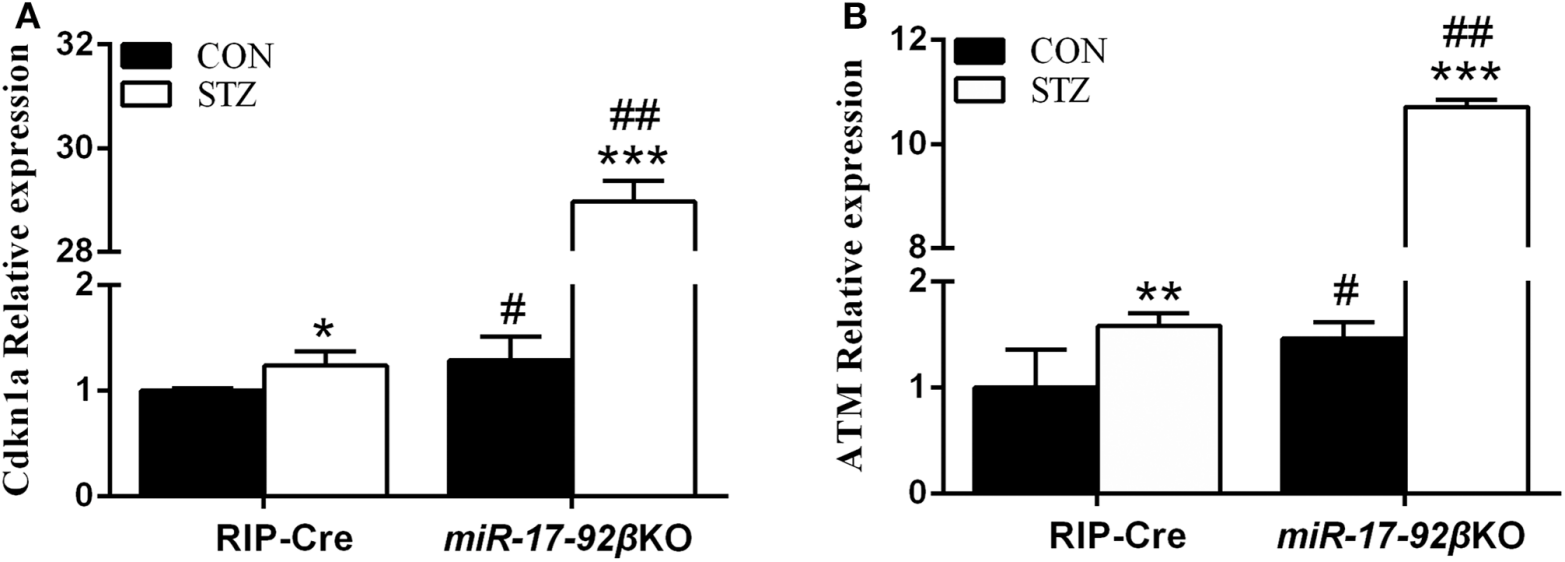
Different expression profiles of genes-related to DNA biosynthesis and damage repair in islets from two genotypes of mice after STZ intervention. **(A,B)** Quantitative RT-PCR illustrated high expression levels of *Cdkn1a* and ATM kinase in isolated islets of *miR-17-92*βKO mice treated with STZ. Significant differences were shown as *P < 0.05, **P < 0.01, ***P < 0.001 compared to the same genotype, #*P* < 0.05, ##*P* < 0.01 compared to the same treatment. *N* = 16–20 mice/group.

As mentioned above, MLD-STZ intervention leads to both impaired glucose tolerance and hyperglycemia and simulates human type 1 diabetes partially through direct beta-cell DNA damage. To determine whether miR-17-92 is involved in beta-cell DNA damage repair, we explored the expression profiles of some identified target genes of STZ and related to DNA synthesis and damage repair in islets from four groups of mice. Compared with RIP-Cre-CON mice, the expression of *Cdkn1a* which inhibit DNA synthesis while promoting DNA damage repair and ATM (ataxia telangiectasia mutated) kinase that is the key enzyme of DNA damage repair up-regulated by 29 and 46%, respectively, in *miR-17-92*βKO-CON mice (Figures 5A,B). Furthermore, the expression levels of *Cdkn1a* and ATM kinase upregulated by 24 and 59% in RIP-Cre-STZ mice, the same as previous studies (41–43), and 2,144 and 631% in islets of *miR-17-92*βKO-STZ mice (Figures 5A,B), leading to cell cycle arrest and DNA synthesis inhibition, but at the same time promotes the repair of damaged DNA, improves the effectiveness of homologous recombinant DNA repair, renovates STZ-induced beta-cell damage, restores the function of damaged beta-cells, and regulates the regeneration and compensation of islet beta-cells for STZ-induced damage. Taken together, these results imply that the miR-17-92 cluster is pivotal for beta-cell adaptation to STZ treatment.

## Discussion

In the present study, the RBG levels of the two genotypes were higher than 300 mg/dl (16.7 mmol/l) after STZ intervention, indicating the diabetic model was successful. Our previous study has revealed high expression levels of miR-17-92 cluster in mouse islets and beta-cell line (30), indicating a significant role of miR-17-92 cluster in normal beta-cell function. In the current study, we found the expression levels of miR-17-92 cluster were elevated to different extent in islets from RIP-Cre-STZ mice, suggesting that the miR-17-92 cluster may be involved in the adaptive response of islet beta-cells to STZ-induced injury.

Consistent with our previous study, the body weight, RBG, and fasting blood glucose of *miR-17-92*βKO-CON mice were similar to those in the RIP-Cre-CON mice (30), implying that there might be other mechanisms in islet beta-cells that coordinated with the miR-17-92 signaling pathway to regulate glucose metabolism, which could partially rectify the abnormal glucose metabolism induced by the homozygous deletion of miR-17-92 cluster. However, after STZ treatment, the *miR-17-92*βKO mice showed more pronounced metabolic disturbance characterized with more obvious body weight loss, higher random and fasting blood glucose levels and more serious glucose intolerance. Histomorphological analysis showed that STZ intervention impaired the morphology and structure of islets in two genotypes of mice. Meanwhile, insulin-glucagon immunofluorescent staining revealed that both RIP-Cre-STZ and *miR-17-92*βKO-STZ mice showed reduced number and total mass of islet beta-cells, which is more severe in islets from the latter group, suggesting the islet beta-cells in two genotypes of mice were in decompensation stage. Taken together, the above results suggest that conditional deletion of miR-17-92 cluster in islet beta-cells reduces their adaptation ability to stress stimulation, and MLD-STZ intervention further deteriorates the function of islet beta-cells, so as to result in disturbance of glucose homeostasis. Our data indicate that miR-17-92 cluster is involved in the adaptive response of islet beta-cells to STZ treatment.

Additionally, previous studies have shown that the number of islet beta-cell fluctuates. Physiologically, the body can adjust the number of islet beta-cells according to the changes of the internal and external environment along with the functional status of beta-cells, so as to maintain a relatively stable blood glucose levels to adapt to the environmental variation (44). The pancreatic insulin-producing beta-cells are derived from the differentiation process during embryo development or from replication which appears postnatally (45, 46), and it is essential to maintain sufficient beta-cell number to respond to aging and metabolic stresses. Limited beta-cell proliferation is extensive in human type 1 and type 2 diabetes. Studies have shown that miR-17-92 targets a series of cell proliferation-related genes, such as *Cdkn1a, p57* (47), and apoptosis-related genes including *Pten, Bcl2L11* (48) to modulate cell proliferation and apoptosis. Nevertheless, the regulation of proliferation and apoptosis of islet beta-cells by miR-17-92 cluster remains largely unclear. Recent studies have found that lipid phosphatase encoded by *Pten*, is a potent negative regulator of phosphoinositide 3-kinase (PI3K)-Akt signaling pathway, which plays an important role in cell proliferation and insulin biosynthesis and secretion (49). Importantly, *Pten* is known to be the target gene of miR-19a and miR-19b-1 (50). *Pten* is also a critical determinant of body size and glucose metabolism in mice (51). Studies have demonstrated that conditional deletion of *Pten* in insulin-producing cells during mouse pancreatic embryonic development (E17.5) or in adult beta-cells significantly increased islet mass and beta-cell proliferation (49), and exerted protective effects against high-fat diet feeding and STZ-induced diabetes (52, 53). In short, *Pten* is a critical negative effector of both beta-cell mass and function, and its expression was up-regulated in diabetic animal models (53). Previous studies have suggested that deletion of miR-17-92 cluster in beta-cells led to impaired glucose tolerance and reduced first-phase insulin secretion, which may be partly mediated by *Pten*-Akt signaling pathway (24, 30). Moreover, in the present study we found higher expression level of *Pten* in beta-cells from both genotypes of mice given STZ intervention, which was obvious in the *miR-17-92*βKO-STZ mice as the synergistic effect of conditional deletion of miR-17-92 cluster and the STZ treatment. Further studies showed that STZ treatment reduced proliferation and increased apoptosis in islet beta-cells in two genotypes of mice, which may be achieved by regulating the expression of *Pten*. It also indicates that STZ intervention decompensated islet beta-cells of two genotypes of mice and resulted in a low transformation status of the beta-cells particularly in the *miR-17-92*βKO-STZ group.

Diabetes mellitus shows features of hyperglycemia because of absolutely or relatively insufficient serum insulin secretion. Pancreatic beta-cells have fundamental functions to modulate insulin synthesis and secretion, which is regulated by various transcriptional activators such as Pdx-1 (pancreas/duodenum homeobox factor-1) (54), MafA (55, 56), and Glut2 (57–59) and transcriptional repressors including Sox6 (60) and Crem (61). Pdx-1 plays pivotal roles in the early differentiation, maturation and regeneration of pancreatic islet cells (57). MafA is highly expressed in pancreatic beta-cells, which activates the transcription of the insulin gene by targeting RIPE3b1, a cis-regulatory element (62–64). The expression of MafA gene is down-regulated, thus inhibiting the transcription of insulin in diabetes mellitus (55, 56). MafA can regulate the expression of insulin gene as well as other related factors including Pdx-1 and Glut2 (57). Glut2 is a glucose transporter in pancreatic beta-cells, which can transport glucose and regulate ion channels on cell membrane, thus promoting insulin secretion (57). Sox6, a member of the high mobility group box superfamily, acts as a co-repressor with Pdx-1 and plays critical roles in pancreatic beta-cell replication and insulin gene transcription (65). Over-expression of Sox6 is involved in decreased insulin gene transcription through inhibition of the activation of insulin gene promoter by Pdx-1 (60). In addition, Crem has also been reported to directly repress the insulin gene promoter activation in beta-cell (66). In the present study, the expression of MafA and Glut2 in islets of RIP-Cre-STZ and *miR-17-92*βKO-STZ group was down-regulated, which reduced insulin gene transcription as previous studies (57–59, 67). Meanwhile, the upregulation of Sox6 and Crem resulted in the decrease of insulin transcription. In summary, the downregulation of MafA and Glut2 along with the upregulation of Sox6 and Crem may be related to the decrease of the transcription of insulin gene and islet beta-cell proliferation in the STZ group. Nevertheless, there is no literature on the relationship between the miR-17-92 cluster and the above factors, which needs further studies.

STZ is a kind of natural compounds produced by streptococcus that specifically damages pancreatic islet beta-cells in mammals. The underlying mechanism is to form isocyanate compounds *in vivo*, which bind to nucleic acid proteins to inhibit the activity of DNA polymerase, and interfere with the synthesis and damage repair of DNA, thus to regulate pancreatic beta-cell proliferation and apoptosis (68). *Cdkn1a* encodes a potential cyclin-dependent kinase inhibitor which represses the activity of cyclin-cyclin-dependent kinase 2/4, then modulates G1 cell cycle progression. The encoded protein also can interact with PCNA (proliferating cell nuclear antigen), a kind of cofactor of DNA polymerase, and regulate DNA replication and damage repair in S cell cycle. Additionally, mice lack of *Cdkn1a* have the ability to regenerate damaged tissue. Besides, ATM kinase increases the phosphorylation levels of H2AX and 53BP1, thus promoting DNA damage repair, improving the effectiveness of homologous recombinant DNA repair to inhibit beta-cell proliferation and reduce their apoptosis. Conditional deletion of the master DNA repair gene-ATM kinase in mouse pancreatic beta-cells protects mice against STZ-induced diabetes (69). Importantly, it has been reported that *Cdkn1a* and ATM kinase are involved in DNA damage repair process in islet beta-cells induced by STZ, and the expression of *Cdkn1a* and ATM kinase were up-regulated after STZ intervention (42, 43, 70). Moreover, studies have found that miR-17 and miR-92a-1 could inhibit *Cdkn1a*, and miR-18a targets ATM kinase thus promoting DNA synthesis and inhibiting DNA damage repair (71, 72). In the current study, the expression of *Cdkn1a* and ATM kinase was up-regulated in *miR-17-92*βKO-CON mice, which was similar as previous studies (71, 72). Interestingly, the expression of *Cdkn1a* and ATM kinase were further up-regulated after STZ intervention in two genotypes of mice (42, 43, 70), which resulted in cell cycle arrest and DNA synthesis repression. At the same time, it promotes the repairment of damaged DNA induced by STZ, restores the function of damaged beta-cells thereby modulating the adaptation of islet beta-cells after STZ-induced injury.

Last but not least, our studies found high epididymal fat pad content in *miR-17-92*βKO-CON mice compared with control mice (30). However, STZ treatment led to the low epididymal fat content in both genotypes of mice, possibly due to the more deteriorated metabolic disturbance. Therefore, *miR-17-92*βKO mice showed lower body weight, higher glucose levels, and more epididymal fat content after STZ treatment, indicating *miR-17-92*βKO mice may be a more suitable animal model to develop diabetes induced by STZ treatment compared with control mice.

In conclusion, our results showed that mice with conditional deletion of the miR-17-92 cluster in pancreatic beta-cells exerted profound metabolic abnormalities due to beta-cell dysfunction and reduced beta-cell proliferation after STZ treatment, indicating that the miR-17-92 cluster is essential for pancreatic beta-cell restoration and adaptation in a chemical-induced diabetes animal model.

## Data Availability Statement

All datasets generated for this study are included in the article/supplementary material.

## Ethics Statement

The animal study was reviewed and approved by the Institute's Animal Care and Use Committee of the West China Hospital.

## Author Contributions

XY designed this research. SW, JL, YM, LL, FC, and JZ were responsible for the experiments. Among them, LL, FC, and JZ were mainly responsible for the histopathological part. The other three were in charge of the animal experiments and the next molecular experiments after the animal were sacrificed. XC, LT, and XY are responsible for the revision of the whole article.

### Conflict of Interest

The authors declare that the research was conducted in the absence of any commercial or financial relationships that could be construed as a potential conflict of interest.
